# Specific targeting of the GABA-A receptor α5 subtype by a selective inverse agonist restores cognitive deficits in Down syndrome mice

**DOI:** 10.1177/0269881111405366

**Published:** 2011-08

**Authors:** J Braudeau, B Delatour, A Duchon, P Lopes Pereira, L Dauphinot, F de Chaumont, J-C Olivo-Marin, RH Dodd, Y Hérault, M-C Potier

**Affiliations:** 1 Centre de Recherche de l'Institut du Cerveau et de Moelle Epinière, CNRS UMR7225, INSERM UMRS 975, UPMC, Paris, France.; 2 CNRS, Lab NAMC, UMR8620, Université Paris Sud, Orsay, France.; 3 Institut de Génétique et de Biologie Moléculaire et Cellulaire (IGBMC), Institut National de Santé et de Recherche Médicale (INSERM) U964/Centre National de Recherche Scientifique (CNRS) UMR 1704/Université de Strasbourg, 67404 Illkirch, France.; 4 Institut Clinique de la Souris (ICS), Groupe d'Intérêt Economique Centre Européen de Recherche en Biologie et en Médecine (GIE-CERBM), INSERM, CNRS, Université de Strasbourg, Illkirch, France.; 5 Institut Pasteur, Quantitative Image Analysis Unit, CNRS URA 2582, 75015 Paris, France.; 6 Institut de Chimie des Substances Naturelles - CNRS UPR 2301, Gif-sur-Yvette, France.

**Keywords:** Down syndrome, GABA-A, inverse agonist, learning, memory, therapy

## Abstract

An imbalance between inhibitory and excitatory neurotransmission has been proposed to contribute to altered brain function in individuals with Down syndrome (DS). Gamma-aminobutyric acid (GABA) is the major inhibitory neurotransmitter in the central nervous system and accordingly treatment with GABA-A antagonists can efficiently restore cognitive functions of Ts65Dn mice, a genetic model for DS. However, GABA-A antagonists are also convulsant which preclude their use for therapeutic intervention in DS individuals. Here, we have evaluated safer strategies to release GABAergic inhibition using a GABA-A-benzodiazepine receptor inverse agonist selective for the α5-subtype (α5IA). We demonstrate that α5IA restores learning and memory functions of Ts65Dn mice in the novel-object recognition and in the Morris water maze tasks. Furthermore, we show that following behavioural stimulation, α5IA enhances learning-evoked immediate early gene products in specific brain regions involved in cognition. Importantly, acute and chronic treatments with α5IA do not induce any convulsant or anxiogenic effects that are associated with GABA-A antagonists or non-selective inverse agonists of the GABA-A-benzodiazepine receptors. Finally, chronic treatment with α5IA did not induce histological alterations in the brain, liver and kidney of mice. Our results suggest that non-convulsant α5-selective GABA-A inverse agonists could improve learning and memory deficits in DS individuals.

## Introduction

Down syndrome (DS) is the consequence of trisomy 21, the most common genetic cause of mental retardation (1/800 live births), and is characterized by varying degrees of cognitive impairments (Sherman et al., 2007). Advances in teaching methods and educational mainstreaming have proven to be beneficial to people with DS, but are clearly not sufficient to counteract all cognitive deficits (Wishart et al., 2007). Since these individuals now have a life expectancy of 55 years and often survive their parents, treatments aimed at enhancing cognitive skills to provide higher autonomy are long-awaited. Unfortunately, attempts with off-label use of various drugs have not been successful (Reeves and Garner, 2007; Wiseman et al., 2009).

Recent data strongly suggest that changes associated with learning and memory dysfunction in DS might result, in part, from defects in the hippocampus associated with increased inhibition (GABAergic activity) in the brain, opening new avenues for pharmalogical intervention (Best et al., 2007; Kleschevnikov et al., 2004). As a consequence, treatment of DS mouse models with non-competitive GABA-A antagonists, such as picrotoxin or pentylenetetrazol, can restore impaired phenotypes in DS mice (Fernandez et al., 2007; Rueda et al., 2008). However, these drugs are convulsant at high doses, precluding their use as cognition enhancers in humans, particularly considering that DS patients are more prone to convulsions (Menendez, 2005). Frequency of seizures has been reported to reach 6–17% in DS people (Veall, 1974) with a triphasic distribution of seizure onset depending on age (infancy, early adulthood and late onset) (Pueschel et al., 1991).

As an alternative to GABA-A antagonists, we searched among ligands of the GABA-A-benzodiazepine receptors that could decrease GABAergic transmission without inducing convulsant activity. This selective pharmacological profile can be obtained using molecules that are active at the α5 subunit-containing GABA-A_-_benzodiazepine receptors (Sur et al., 1999). These receptors are largely expressed in the hippocampus (Wisden et al., 1992), an area integral to learning and memory. Molecules that specifically decrease GABAergic transmission through these receptors, such as α5-selective inverse agonists, have been shown to enhance cognition and synaptic plasticity without having any adverse convulsant/pro-convulsant or anxiogenic effects (Ballard et al., 2009; Collinson et al., 2006; Dawson et al., 2006). To the best of the authors’ knowledge, these compounds have not yet been evaluated for the treatment of cognitive impairments associated with brain dysfunction.

The goal of the present work was to assess the therapeutic potential of an α5-selective inverse agonist, the orally active 3-(5-methylisoxazol-3-yl)-6-[(1-methyl-1, 2, 3-triazol-4-yl)methyloxy]-1,2,4-triazolo[3,4-a]phthalazine (Sternfeld et al., 2004), referred to herein as compound α5IA, in cognitively impaired mouse models of DS. We used Ts65Dn mice, which are trisomic for orthologues of about half of the genes on human chromosome 21 (Reeves et al., 1995). These mice demonstrate learning and memory defects, as well as synaptic plasticity abnormalities and are widely used for preclinical research on DS (Escorihuela et al., 1995; Kleschevnikov et al., 2004; Reeves et al., 1995).

## Materials and methods

### Animals

Male mice were produced at the Intragene resource centre (TAAM, CNRS UPS44 Orléans, France) and bred on a mixed genetic background B6C3, derived from C57BL/6J (B6) and a congenic inbred line C3H/HeH for the BALB/c wild-type *Pde6b* allele (Hoelter et al., 2008), thus avoiding retinal degeneration and impaired visual acuity. On this background, Ts65Dn mice show similar behavioural phenotypes when compared with the original Ts65Dn line (AD and YH, personal communication; see also Costa et al., 2010). Mice were acclimated in our animal facility for at least 2 weeks before initiating behavioural testing. For each experiment, different batches of mice (3 months old) were used (total number of animals used: Ts65Dn mice, *n* = 90; euploid littermates, *n* = 122).

All experiments were conducted in accordance with the ethical standards of French and European regulations (European Communities Council Directive of 24 November 1986). The supervisor of *in vivo* studies (B Delatour) received official authorization from the French Ministry of Agriculture to carry out research and experiments on animals (authorization number 91-282).

### Real-time quantitative PCR of Gabra-5

Total RNA was extracted from dissected hippocampi of nine euploid and seven Ts65Dn mice and treated with DNase using the Nucleospin RNA II kit (Macherey-Nagel, France). RNAs (500 ng) were individually reverse-transcribed into cDNAs overnight at 37°C using the Verso cDNA kit (ThermoFisher Scientific, Waltham, USA) according to the manufacturer's instructions. qPCR assays were performed in a Lightcycler® 480 System (Roche), in the presence of 200nM of each primer (*Gabra5* 5’gacggactcttggatggcta3’_forward and 5’acctgcgtgattcgctct3’_reverse; *pPib* 5’ttcttcataaccacagtcaagacc3’_forward and 5’accttccgtaccacatccat3’_reverse for normalization), 100nM of specific hydrolysis probe and 1X Lightcycler® 480 Probes Master mix (Roche, France), and normalized using the Lightcycler® 480 SW 1.5 software.

### α5IA synthesis and formulation

The drug used was 3-(5-methylisoxazol-3-yl)-6-[(1-methyl-1,2,3-triazol-4-yl)methyloxy]-1, 2, 4-triazolo[3, 4-a]phthalazine (α5IA). It was synthesized by Orga-Link SARL (Magny-les-Hameaux, France), according to Sternfeld et al. (2004). The hydrochloride salt was prepared by dissolving the base in hot ethanol and adding a solution of 5% hydrochloric acid in ethanol until the solution was slightly acidic. Upon cooling, a precipitate formed which was collected by filtration, washed with cold ethanol and dried.

The HCl salt of α5IA was solubilized in a mixture of DMSO, Cremophor El (BASF, Ludwigshafen, Germany) and hypotonic water (ProAmp®) (10:15:75). α5IA or vehicle (solubilization solution) was injected intraperitoneally (i.p.) at different doses ranging from 1 to 50 mg/kg.

### Morris water maze

Experiments were performed in a 150-cm diameter Morris water maze filled with opacified water kept at 19°C and equipped with a 9 cm diameter platform submerged 1 cm under the water surface.

**Figure 1.**
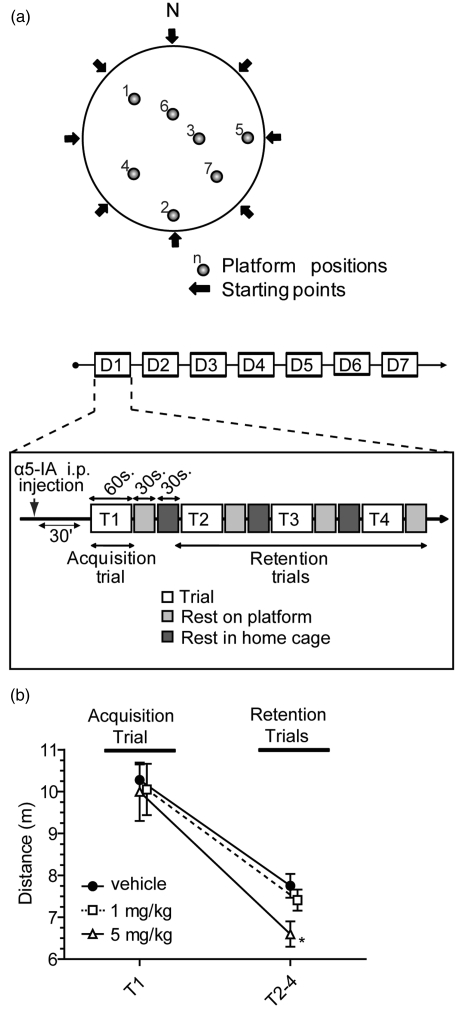
Dose–response effect of α5IA. The optimal α5IA promnesic dose was determined in euploid mice trained in the DMTP task. (a) Schematic representation of the DMTP protocol. Training was performed during 7 consecutive days. Each day, mice underwent one acquisition trial (T1) and three retention trials (T2–T4); inter-trial interval was 60 seconds. The position of the platform was changed every day, but remained constant within each session. (b) Performance (distance to platform; mean ± SEM) of the mice between acquisition and retention trials. Data from the seven training days have been pooled. All mice showed a significant increase in behavioural accuracy within each session. While vehicle-treated mice and mice receiving 1 mg/kg of α5IA showed similar retention, mice that were treated with the 5 mg/kg dose of α5IA displayed a significantly higher retention performance in comparison to other groups (*p < 0.05, ANOVA with Fisher's *post hoc* comparisons).

In a first pilot experiment, a total of 27 C57BL/6 mice were used to study dose–response cognitive-enhancement effects of α5IA (vehicle, 1 mg/kg, 5 mg/kg, *n* = 9 in each group) in a delayed matching-to-place task (DMTP) (see Figure 1(a)) as described previously (Collinson et al., 2002).

**Figure 2.**
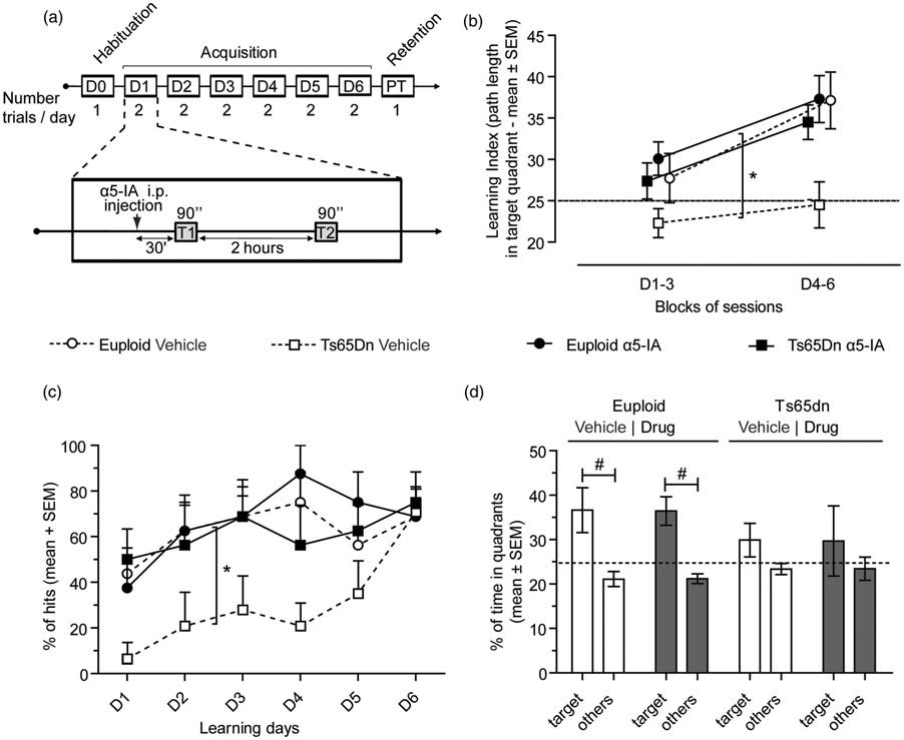
α5IA restores spatial learning in Ts65Dn mice. (a) Schematic representation of the MWM protocol (see the text for explanations; PT: probe trial). (b) Data on learning performance have been pooled into two blocks of 3 days. Vehicle-treated Ts65Dn mice demonstrated decreased learning index in comparison with the three other trained groups. This deficit was corrected by treatment with α5IA. (c) A hit was defined as reaching the platform before 90 seconds. Vehicle-treated Ts65Dn mice showed a clear delay in conditioning that was rescued after treatment with α5IA. (d) Only euploid mice showed a spatial bias for the platform target quadrant during probe trial. Impaired retention of the platform location in Ts65Dn mice was not rescued after drug treatment. For (b) and (d), horizontal dotted lines at 25% correspond to random performance. For (d), percentage of time spent in other quadrants (‘others’) calculated as the mean time spent in these three non-target quadrants. *p < 0.05: ANOVA with repeated measures and contrast analysis. #*p* < 0.05: paired Student's *t*-tests.

In a second experiment, 16 Ts65Dn (vehicle *n* = 8, α5IA 5 mg/kg *n* = 8) mice and 16 euploid littermates (vehicle *n* = 8, α5IA 5 mg/kg *n* = 8) were trained during 6 days in the standard Morris water maze task (MWM) (see Figure 2(a)). Training consisted of daily sessions (two trials per session). Start positions varied pseudo-randomly among the four cardinal points. Mean inter-trial interval was 2 hours. During the habituation and spatial training phases each trial ended when the animal reached the platform. A 90-second cut-off was used, after which mice were manually guided to the platform. Once on the platform, animals were given a 20-second rest before being returned to their cage. Twenty four hours after the last training trial, retention was assessed during a probe trial in which the platform was no longer available. During the four subsequent sessions visual ability of mice was controlled: platform location was cued by a white styrene ball placed 12 cm above water surface and access to external indices was prevented by a black curtain surrounding the pool.

In all navigation tasks (DMTP, MWM) mice were injected daily with vehicle or α5IA 30 min before each first (T1) trial of each daily session. Animals were monitored with the Any-Maze (DMTP task; Stoelting, Wood Dale, USA) or the VideoTrack (MWM task; Viewpoint, Lyon, France) video analysis systems.

### Novel-object recognition

The apparatus consisted of a square open field (50 cm × 50 cm) placed in a room with weak controlled luminosity (4–6 lux) and constant 60 dB white noise.

The first day, all animals (16 euploid and 16 Ts65Dn mice) were handled by the experimenter. On day 2, mice were habituated for 20 min to the empty arena. On day 3, four identical objects were placed symmetrically 14 cm away from the arena corners. Mice were free to explore the objects for 20 min. On the test day (day 4), mice were injected i.p. with either vehicle or α5IA 5 mg/kg (eight euploid and eight Ts65Dn mice in each group). Thirty minutes after injections, mice were placed in the arena containing two identical objects, and allowed to explore them for 10 min. Mice then returned to their home cage for a 10-min retention interval. To test short-term recognition memory, one familiar object and one novel object were placed in the apparatus, and mice were free to explore for a 10-min period. Between each trial the arena and objects were cleaned with 70° ethanol to reduce olfactory cues.

**Figure 3.**
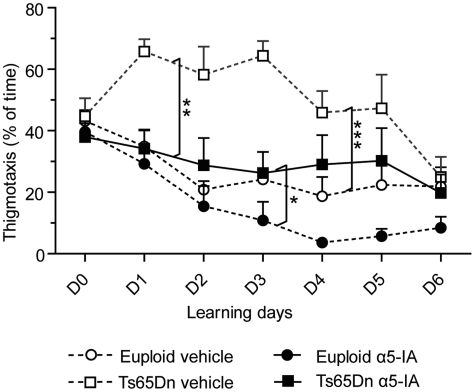
α5IA relieves the use of inadequate behavioural navigating strategies in the Morris water maze. A robust effect of α5IA was observed on thigmotaxy (percentage of time spent performing thigmotaxy has been pooled over the six training sessions, mean ± SEM). This inadequate strategy to locate the platform in the water maze was strongly decreased following treatment with α5IA but more particularly in Ts65Dn mice and to a lesser (non-significant) extent in euploid mice, likely due to some ceiling effects as Ts65Dn mice displayed an overall increased basal level of thigmotaxy in comparison to euploid mice. *p < 0.05; **p < 0.001; ***p < 0.0001; ANOVA with repeated measures and contrast analysis.

During all sessions mice were monitored using the Any-Maze video-tracking software. Object exploration was manually scored with an ethological keyboard and defined as the orientation of the nose to the object at a distance <4 cm. The amount of time exploring familiar vs. novel objects was calculated to assess memory performance.

### Measure of cerebral Fos immunoreactivity

Euploid (*n* = 13) and Ts65Dn (*n* = 6) mice were pseudo-trained in the object recognition task using the same protocol as described in the novel-object recognition (NOR) task, but with no retention phase. Thirty minutes before acquisition, six euploid and three Ts65Dn mice, and seven euploid and three Ts65Dn were injected i.p. with α5IA (5 mg/kg) or vehicle, respectively. Following the acquisition session, mice returned to their home cage. Ninety minutes following behavioural stimulation, mice were perfused transcardially with phosphate buffered saline (PBS), their brains fixed in 10% formalin, cryoprotected and sectioned on a freezing microtome. Fos immunoreactivity (polyclonal AB-5, Calbiochem-VWR, France; dilution 1:10,000) was detected using the ABC method with nickel-enhanced diaminobenzidine as final chromogen. Immunoreactivity was quantified using QUIA software (see http://www.bioimageanalysis.org) that automatically calculated the proportion of stained tissue (*p* = stained area/total area), providing unbiased stereological measurements. Four regions of interest (ROIs) were analysed: posterior cingulate cortex, perirhinal cortex, dentate gyrus and CA1 field of the hippocampus on several serial sections. Results were then averaged to give a reliable quantitative evaluation of local Fos immunostaining.

### Convulsant and pro-convulsant effects

The convulsant effects were evaluated after a single i.p. injection of α5IA at high dosage (50 mg/kg) or vehicle in Ts65Dn or euploid littermates. For testing the pro-convulsant effects of α5IA, a sub-convulsant dose of pentylenetetrazole (45 mg/kg i.p.) was injected i.p. 20 min after injection of α5IA or vehicle. Six or seven mice were used for each condition. Mice were observed for 20 min (convulsant effects) or 30 min (pro-convulsant effects): the occurrence of tonic convulsions and latency to the first myoclonic jerk episode were recorded.

### Locomotor activity

Locomotor activity was evaluated in a total of 33 mice 30 min after i.p. injections (vehicle: 8 euploid and 7 Ts65Dn mice; α5IA 5 mg/kg: 10 euploid and 8 Ts65Dn mice). Locomotion was measured in a square open field (50 cm × 50 cm; luminosity: 30 lux) with black walls 30 cm high. Each animal was allowed to freely explore the arena for 10 min. Horizontal activity was monitored using the Any-Maze software. Time spent in the 10-cm wide peripheral zone and in the complementary 30 cm × 30 cm central zone was recorded to evaluate anxiety.

### Anxiety-related behavioural testing

Modulation of anxiety-related behaviours by α5IA was assessed using an elevated plus maze, in a total of 42 mice, 30 min after i.p. injections (vehicle: 11 euploid and 7 Ts65Dn mice; α5IA 15 mg/kg: 14 euploid and 10 Ts65Dn mice). The maze was constructed of black Perspex (length, 28 cm; width, 5 cm; height from floor, 40 cm; overall luminosity in open arms: 70 lux) with two opposing open arms, and two enclosed arms equipped with three 16-cm high walls. Mice were placed in the central region of the maze and behaviour was recorded for a 5-min period using the Any-Maze software.

To explore the potential adversity of chronic injections of α5IA, another group of euploid mice was treated for 2 weeks (5 mg/kg, five injections/week; five α5IA-treated mice; five vehicle-treated mice) and then evaluated in the elevated plus maze as described previously.

### Anatomopathology after chronic treatment with α5IA

Mice treated for 2 weeks with α5IA 5 mg/kg and tested in the elevated plus maze (see the previous section) were further treated for another 3 weeks. On the last day of treatment, urine samples were collected 2 hours after α5IA or vehicle i.p. administration. The next day, mice were sacrificed. For anatomo-pathological examination, three additional euploid non-injected mice were also sacrificed. Liver, kidney, brain and spleen were dissected and fixed in a 10% formalin solution. Tissues were then paraffin-embedded, cut and processed for routine histopathological evaluation (haematoxylin–eosin and periodic acid-Schiff stainings).

### Statistical analysis

In most cases, data were analysed using an analysis of variance (ANOVA) with Fisher's *post hoc* comparisons. ANOVA with repeated measures or within-subjects designs and contrast analysis were carried out when required by the experimental plan to assess complementary statistical effects. Also in some designs, statistical analysis was performed using Student's *t*-tests. For all analysis statistical significance was set to a *p*-value <0.05. All analyses were performed using Statistica v6 (StatSoft, Inc., Tulsa, OK, USA) or GraphPad Prism (GraphPad Software, La Jolla, CA, USA) software.

## Results

### α5IA acts as a cognition enhancer and alleviates learning and memory deficits in Ts65Dn mice

#### Synthesis of the α5IA and determination of the pharmacologically active dose

As a prerequisite we checked that the level of expression of the *Gabra5* gene encoding the α5 GABA-A subunit was unchanged in the hippocampus of Ts65Dn mice as compared with euploids (*t*_14_ = 0.40, *p* = 0.69; data not shown) confirming the presence of the pharmacological target in Ts65Dn mice. We concurrently synthesized α5IA and showed that the spectral characteristics and binding affinity of the compound conformed with published data (see Supplementary Figure S1) (Sternfeld et al., 2004). We then determined the optimal dose of α5IA that induced clear promnesic effects in mice trained in the DMTP version of the MWM task (Figure 1(a)). As illustrated in Figure 1(b), a large decrease in distance travelled was observed between acquisition and retention trials underlying memory of the goal location (*F*_1,24_ = 66.39, *p* < 0.0001). The three groups (vehicle, α5IA 1 mg/kg and α5IA 5 mg/kg) showed similar performances during acquisition (*F* < 1) but a group effect was observed during retention trial (*F*_2,24_ = 4.5, *p* < 0.05). Indeed while the vehicle and the α5IA 1 mg/kg groups demonstrated comparable retention performance (*F* < 1), mice treated with α5IA 5 mg/kg displayed a clear improvement of performance (comparison with vehicle mice: *F*_1,24_ = 8.5, *p* < 0.01). We therefore selected the dose of 5 mg/kg to be used in subsequent behavioural tests in DS models.

#### Effects of α5IA on reference memory in Ts65Dn mice using the Morris water maze task

**Figure 4.**
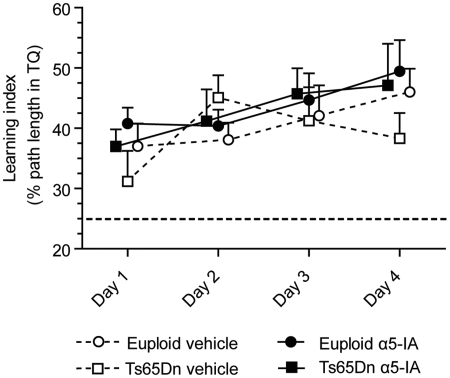
Spatial impairments in Ts65Dn mice are not due to visual deficits. Following evaluation of spatial memory in the Morris water maze (MWM), mice were trained in a visually guided navigation task (cued visible platform). Performance was assessed using an unbiased learning index (mean ± SEM, same as in Figure 2). Analysis indicated that behavioural accuracy to locate the visible platform increased across sessions with no effect of genotype or treatment. The horizontal dotted line at 25% represents level of performance due to random navigation in the pool. As illustrated, all trained groups performed largely above this level.

To evaluate the rescuing potential of α5IA in behaviourally impaired Ts65Dn mice, we first assessed the effect of the drug on spatial reference memory in the standard MWM task, in which mice have to swim in their environment to locate a hidden platform at a constant location (Figures 2–4). Out of 16 Ts65Dn and 16 euploid mice, 1 Ts65Dn mouse was discarded from statistical analysis because it displayed abnormal floating behaviour and decreased swim speed in the maze. During the probe trial one euploid mouse was removed from the analysis for the same reason.

We first analysed the acquisition of place location (Figure 2(b) and 2(c)). ANOVA on swim speeds revealed an effect of group factor (*F*_3,26_ = 2.99, *p* < 0.05). Owing to variations in swim speeds between conditions that may impact non-specifically on performances, we calculated an unbiased index of spatial learning that is the percentage of the path length spent by mice in the target quadrant (Faure et al., 2009; Janus et al., 2004) (Figure 2(b)). ANOVA (main factors: group and block of sessions) on this learning index indicated significant effect of group (*F*_3,27_ = 4.77, *p* < 0.01) and block (*F*_1,27_ = 16.63, *p* < 0.001) factors with no significant interactions between these main factors (*F*_3,27_ < 1). Vehicle-treated Ts65Dn mice displayed a low learning index when compared with mice from the three other groups (all *F*_1,27_ > 6.53, *p* < 0.05). ANOVA on the percentage of trials performed within the cut-off limit (that is, percentage of hits, Figure 2(c)), a complementary measure of learning proficiency, indicated significant effect of group (*F*_3,27_ = 3.44, *p* < 0.05) and session (*F*_5,135_ = 4.32, *p* < 0.002) factors with no significant interactions between these main factors (*F*_5,135_ = 0.91, *p* > 0.55). Vehicle-treated Ts65Dn mice were once again severely impaired in terms of hits performed when compared with mice from the three other groups (percentage of hits: all *F*_1,27_ > 6.10, *p* < 0.05, Figure 2(c)). Finally, ANOVA indicated that α5IA significantly potentiated the acquisition proficiency of Ts65Dn mice (*F*_1,27_ > 6.10, *p* < 0.025 for the learning index and hit measures) allowing them to regain normal levels of performance. This promnesic effect of the treatment was not observed in euploid mice that performed equally well in this test with or without α5IA (*F* < 1 for the learning index and percentage of hits measures).

We then analysed navigation strategies of mice (Figure 3). Indeed, in association with an impaired learning capacity, Ts65Dn mice also displayed high levels of thigmotaxy. We measured this ‘wall-seeking behaviour’ as the time spent by mice in the 10-cm-wide peripheral annulus of the pool. As shown in Figure 3 thigmotaxy of Ts65Dn mice appeared to be strongly decreased after α5IA treatment. ANOVA on the time spent performing thigmotaxy confirmed significant effects of group (*F*_3,28_ = 13.70; *p* < 0.0001) and day (*F*_5,140_ = 7.48; *p* < 0.0001), underlining that thigmotaxy decreased across training sessions (Figure 3). The group × day interaction was non-significant (*F*_15,140_ = 1.37, *p* = ns). *Post-hoc* analysis showed that Ts65Dn mice were more thigmotactic in comparison to euploid mice in vehicle condition (*F*_1,28_ = 20.75, *p* < 0.0001). While α5IA strongly reduced thigmotaxy in Ts65Dn mice (*F*_1,28_= 15.74; *p* < 0.001) these mice still displayed increased thigmotaxy after α5IA treatment (comparison with euploids: *F*_1,28_ = 4.81, *p* < 0.05). In addition, in euploid mice, the thigmotaxy-reducing effect of α5IA, although observed (see Figure 3), did not reach statistical significance (*F*_1,28_ = 2.57, *p* = ns).

**Figure 5.**
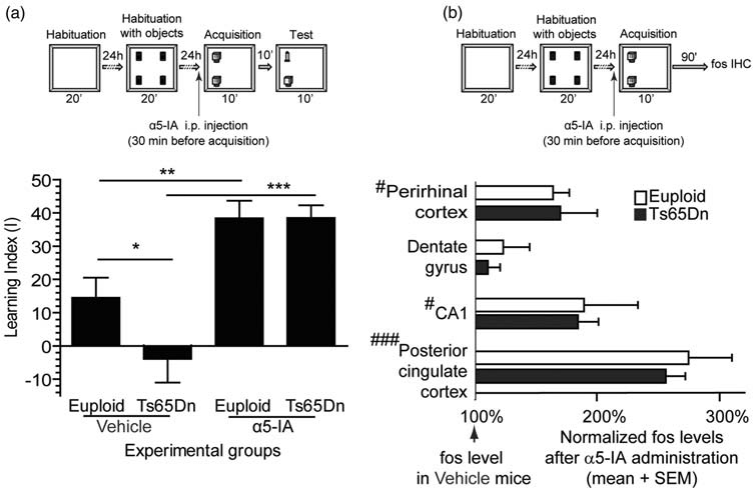
α5IA alleviates recognition memory deficits in Ts65Dn mice and potentiates neuronal activity (a) Upper part: general protocol of the novel-object recognition (NOR) (see the text for explanations). Lower part: Learning index (see Table 1 for raw data). Under vehicle, Ts65Dn mice were found to be impaired. Following i.p. injection of α5IA (5 mg/kg), both euploid and Ts65Dn mice improved their NOR performance and the deficit of Ts65Dn mice was abolished. *p < 0.05; **p < 0.001; ***p < 0.0001; ANOVA with Fisher's *post hoc* comparisons. (b) Upper part: general protocol for assessing the levels of Fos after behavioural stimulation (see the text for explanations). Lower part: histograms depict the relative increase of Fos immunoreactivity in α5IA-treated mice normalized against values obtained for vehicle-treated littermates. In all brain regions sampled, except the dentate gyrus, a significant increase of Fos was observed after α5IA injection. ^#^*p* < 0.05; ^###^*p* < 0.001; two way ANOVA with repeated measures and contrast analysis. No differences between genotypes were observed.

Retention of place location was evaluated during a single probe trial (PT) (no platform available, see Figure 2(a)). Examination of each group separately showed that euploid mice clearly located the target quadrant as demonstrated by their biased exploration (comparison between target vs. non-target quadrants, paired *t*-test: *t*_12_ > 2.91, *p* < 0.025 for vehicle and α5IA conditions; see Figure 2(d)). In contrast, Ts65Dn mice, even after α5IA treatment, did not show exploratory preference for the target quadrant during probe test (*t* < 1.7, *p* = ns for all treatment conditions), indicating that they could not efficiently remember the goal location.

Finally, although mice produced for this study carried a functional allele of Pd6b avoiding retinal degeneration (see the Materials and methods section), their visual ability was controlled using a non-spatial training procedure (Figure 4). ANOVA showed no effects of the group factor (*F*_3,28_ < 1, *p* = ns). The repetition of training trials (day factor) had a significant impact on performance (*F*_3,84_ = 3.11, *p* < 0.05) and there was no group × day interaction (*F* < 1) thus indicating that all groups gradually increased their performance in the visual discrimination task and performed equally, whatever the genotype or treatment.

In summary it can be concluded that α5IA treatment rescued the MWM spatial learning deficits present in Ts65Dn mice and mitigated their use of inadequate navigation strategies.

#### Effects of α5IA on short-term memory in Ts65Dn mice using the novel object recognition task

We then evaluated α5IA treatment effects on non-spatial memory using the NOR paradigm assessing short-term recognition memory (Figure 5(a)).

Out of 16 Ts65Dn and 16 euploid mice, 2 Ts65Dn and 1 euploid were removed from statistical analysis because they displayed abnormally low levels of object exploration (*t* < 7 s) during retention test, hence precluding analysis of their memory performance. The remaining mice spent a large amount of time exploring objects (*t* = 77 ± 4.9 s).

A preliminary analysis of global levels of object exploration was carried out during the acquisition and retention phases of the object recognition task (data not shown). ANOVA did not show any effects of the group (*F*_3,25_ = 2.40, *p* = ns) and testing phase (*F*_1,25_ = 3.67, *p* = ns) nor of the interaction between these factors (*F*_3,25_ < 1, *p* = ns). These results demonstrate that whatever their genotype and treatment, mice displayed the same overall levels of exploration directed towards objects.

Object recognition memory performance was then specifically evaluated during the retention phase by analysing the time spent by mice exploring familiar versus novel objects (Table 1). Unpaired *t*-tests showed that euploid mice, treated or not with α5IA, were able to discriminate between the two objects (vehicle condition: *t*_6_ = 2.49, *p* < 0.05; α5IA condition: *t*_7_ = 6.3, *p* < 0.001) indicating normal recognition memory. In contrast, vehicle-treated Ts65Dn did not show any significant exploratory preference towards the novel object (*t*_7_ < 1) underscoring impaired recognition memory. However, Ts65Dn mice treated with α5IA were able to clearly differentiate between the two objects, indicating that α5IA treatment was able to restore normal recognition memory (*t*_5_ = 4.85, *p* < 0.005).

**Table 1.** α5IA modulates the time spent by mice exploring familiar versus novel objects

Genotype	Treatment	New object mean ± SEM	Familiar object mean ± SEM
Euploids	Vehicle	44.91 ± 6.43	32.43 ± 3.47*
	α5IA (50 mg/kg)	44.40±6.83	28.48 ± 4.16***
Ts65Dn	Vehicle	44.91 ± 5.79	48.35 ± 5.09
	α5IA (50 mg/kg)	49.28 ± 7.27	21.05 ± 2.16***

In contrast to Ts65Dn vehicle-treated mice, vehicle-treated euploids and α5IA treated mice (Ts65Dn and euploids) discriminated between familiar and novel objects. Comparison between objects: *p < 0.05, ***p < 0.001, paired *t*-test.

In order to better clarify the effects of genotype and α5IA treatment on recognition memory we calculated a learning index (I) according to the following formula:
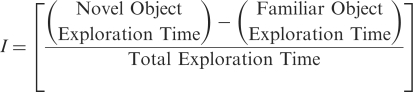
ANOVA on this learning index indicated significant effect of group (*F*_3,25_ = 12.52, *p* < 0.001). *Post-hoc* analysis indicated a significant effect of α5IA treatment which largely potentiated recognition memory (Figure 5(a)). The effect was observed in both euploid (comparison vehicle vs. drug conditions: *F*_1,25_ = 8.42, *p* < 0.01) and Ts65Dn mice (*F*_1,25_ = 24.34, *p* < 0.0001). The analysis also showed that vehicle-treated Ts65Dn mice had a lower learning index as compared with euploid mice (*F*_1,25_ = 4.97, *p* < 0.05). However, following α5IA treatment, the learning index of Ts65Dn and euploid mice were found to be similar (*F* < 1), underlining recovery of performance following treatment in this genotype.

In summary, Ts65Dn mice under vehicle condition presented impaired recognition memory in the NOR task that was recovered, after α5IA treatment.

### α5IA potentiates evoked-neuronal activity

In order to determine how α5IA modulated behaviour-evoked neuronal activity in euploid and Ts65Dn mice, we performed a brain mapping analysis of an immediate early gene product (Fos protein). Animals were trained as described previously in the NOR task until completion of the acquisition phase (Figure 5(b)). We first confirmed that all groups displayed the same level of object exploration, with no effect of genotype, treatment and of their interactions (all *F* < 1; data not shown). In addition, the distance travelled by mice did not vary significantly with genotype (*F* < 1) and treatment (*F*_1,29_ = 2.29, *p* = ns) (data not shown). It was then concluded that all mice received the same sensorimotor stimulation during the acquisition phase of the NOR task. Ninety minutes after completion of behaviour, mice were sacrificed and their brains processed for quantitative assessment of the neuronal activity marker Fos (Figure 5(b)). The proportion of brain tissue immunolabelled against Fos was quantified and analysed using ANOVA. This analysis revealed a significant effect of Treatment as immunoreactivity was found to be significantly increased in α5IA treated mice (*F*_1,13_ = 6.376, *p* < 0.025). There was, however, no effect of the genotype or of the interactions between genotype and treatment (*F* < 1), suggesting that euploid and Ts65Dn mice displayed the same overall levels of Fos immunoreactivity and underwent similar effects after α5IA treatment. Complementary analysis showed that the effect of α5IA was not the same throughout brain regions (*F*_3,39_ = 85.93, *p* < 0.0001; Figure 5(b)) illustrating that NOR-evoked neuronal activity was restricted to some brain areas (CA1, perirhinal and posterior cingulated cortices). The interaction between region and treatment was found to be significant (*F*_3,39_ = 5.612, *p* < 0.005), likely due to the lack of α5IA-induced increase of Fos immunoreactivity in one of the four regions analysed, the dentate gyrus (effect of treatment: posterior cingulate cortex *F*_1,15_ = 35.59, *p* < 0.0001; perirhinal cortex *F*_1,15_ = 6.37; *p* < 0.025; CA1 *F*_1,15_ = 5.30, *p* < 0.05; dendate gyrus *F* < 1).

We thus concluded that following behavioural stimulation, α5IA enhanced evoked immediate early gene products in specific brain regions such as hippocampus, perirhinal and posterior cingulate cortices.

### α5IA treatment does not induce side effects in Ts65Dn and euploid mice

#### Convulsant and pro-convulsant effects

The α5IA molecule was demonstrated previously to be neither convulsant nor anxiogenic in wild-type mice and rats (Dawson et al., 2006); however, this characteristic had never been tested in DS mouse models. We tested the putative convulsant effect of α5IA after a single injection of 50 mg/kg (10× the dose producing promnesic effects). Neither euploid nor Ts65Dn mice displayed any convulsions after injection (Table 2). We then tested the pro-convulsant effect of α5IA by injecting it (50 mg/kg) 20 min before a sub-convulsant dose of pentylenetetrazol (45 mg/kg) that induces myoclonic convulsions in about 50% of mice. Injection of α5IA did not potentiate convulsant activity of pentylenetetrazol in either euploid or Ts65Dn mice (ANOVA on the latency of myoclonic jerks: effect of group *F*_3,8_ = 1.43, *p* = ns).

**Table 2.** Lack of convulsant and pro-convuslant activities of α5IA

Genotype	Treatment	Latency of myoclonic jerks mean ± SEM	Rate of convulsant mice
**Convulsant effects**			
Euploids	Vehicle	/	0/6
	α5IA (50 mg/kg)	/	0/6
Ts65Dn	Vehicle	/	0/6
	α5IA (50 mg/kg)	/	0/7
**Pro-convulsant effects after pentylenetetrazol (45 mg/kg)**			
Euploids	Vehicle	448±145	4/6
	α5IA (50 mg/kg)	330±90	3/6
Ts65Dn	Vehicle	507±39	3/6
	α5IA (50 mg/kg)	796±354	4/7

Data indicate that α5IA (50 mg/kg) did not induce any convulsant effects in either euploid or Ts65Dn mice. The drug also did not promote the convulsant action of pentylenetetrazol (45mg/kg) in the two genotypes.

#### Locomotor activity

**Figure 6.**
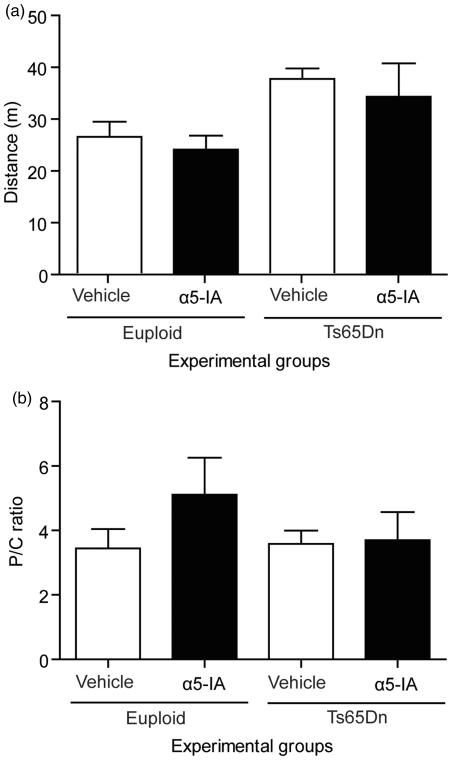
α5IA does not alter locomotor activity and anxiety of Ts65Dn and euploid mice in the open field. Effects of α5IA (5mg/kg) were evaluated on locomotion and anxiety in the open field. (a) Analysis of horizontal activity (travelled distances; mean ± SEM) did not show any effect of treatment, underscoring that a single α5IA injection did not modify the gross locomotor activity of both euploid and Ts65Dn mice. (b) To assess anxiety during the open field session, a periphery-to-centre exploration ratio was measured (P/C ratio; mean ± SEM). Analysis of this measure did not reveal any effects of Genotype or Treatment.

In the open field task, ANOVA on travelled distances (Figure 6(a)) did not show any effect of group (*F*_3,29_ = 2.64, *p* = ns). To evaluate anxiety during the open field test, a periphery-to-centre exploration ratio was measured. ANOVA on this measurement did not reveal any effect of group (*F* < 1; Figure 6(b)).

#### Putative anxiogenic effects

**Figure 7.**
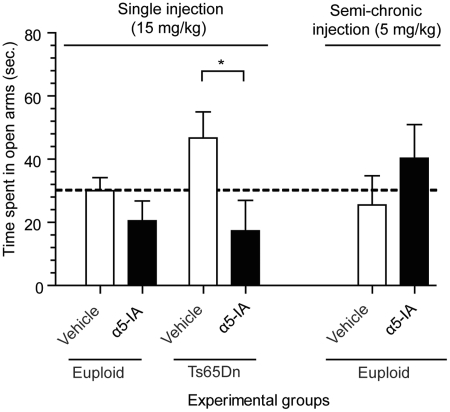
α5IA does not induce anxiety-related behaviours. Anxiety was assessed in the standard elevated plus maze task, in both euploid and Ts65Dn mice under vehicle or α5IA (one single 15 mg/kg i.p. injection; left panel of the figure). Under vehicle condition, Ts65Dn mice showed a trend for hypoanxiety (increased time in open arms) in comparison to euploid mice. Acute treatment with α5IA did not modify the behaviour of euploid mice, but significantly reduced the time spent in open arms by Ts65Dn mice. This effect can be ascribed to a normalization of behaviour in the Ts65Dn mice. Semi-chronic injections of α5IA in euploid mice (5 mg/kg five times a week for 2 weeks; right panel of the figure) did not alter the anxiety levels. Horizontal dotted line indicates the baseline performance of mice acutely treated with vehicle. *p < 0.05, ANOVA with Fisher's *post hoc* comparisons.

In order to better assess the level of anxiety in euploid and Ts65Dn mice treated or not with α5IA, we used the elevated plus maze task. Time spent in the open arms of the elevated plus maze was taken as a measure of anxiety levels (the greater the time spent, the less anxious). ANOVA of this measure did not show any significant effect of group (*F*_3,40_ = 2.59, *p* = 0.06). We nevertheless observed that vehicle-treated Ts65Dn mice had an increased propensity to stay in open arms as compared to euploid mice (*F*_1,40_ = 3.68, *p* = 0.062) and hence displayed some trends for hypo-anxiety traits (for similar findings see Demas et al., 1996). In addition, as illustrated in Figure 7, it appears that α5IA slightly decreased time spent in the open arms. This tendency was significant in Ts65Dn mice (*F*_1,40_ = 4.56, *p* < 0.05) but not in euploid mice (*F* < 1). We therefore propose that the weak ‘anxiogenic-like’ effects of α5IA in Ts65Dn mice are mainly due, in our experimental design, to a normalization of behaviour, from low to normal levels of anxiety, in these mice.

#### Effects of chronic treatment with α5IA

Euploid mice treated with α5IA (5 mg/kg) for 2 weeks did not show any change in their gross behaviour. Body weights were comparable between vehicle and α5IA mice (*F* < 1) and both groups showed normal progressive growth (ANOVA on body weights: *F*_8,64_ = 22.45, *p* < 0.0001, data not shown).

More importantly, mice treated chronically with α5IA 5 mg/kg showed similar levels of anxiety as vehicle-treated mice (unpaired *t*-test on the time spent in open arms: *t*_8_ = 1.04, p = ns; Figure 7, right panel), suggesting that α5IA chronic treatment did not alter anxiety-related behaviours.

**Figure 8.**
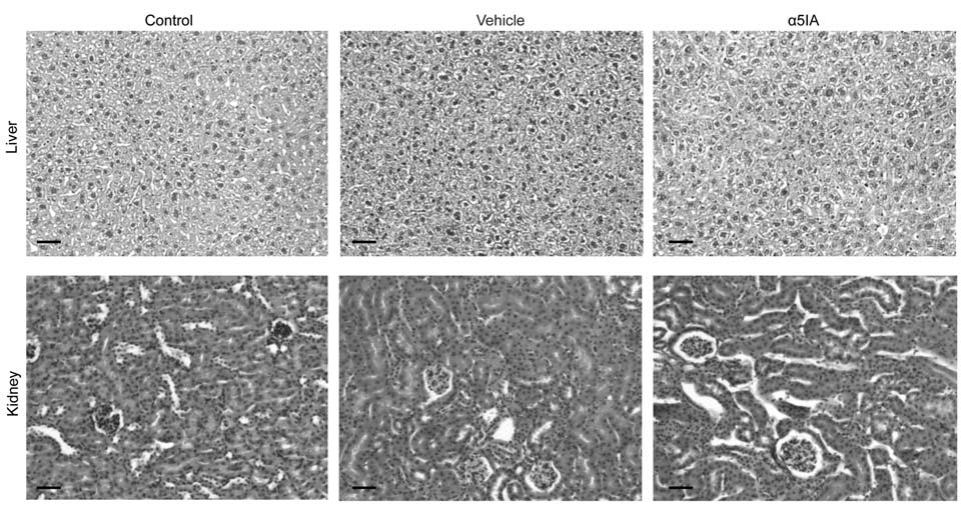
α5IA does not induce any histological lesions after chronic treatment. Following chronic treatment with α5IA (5 mg/kg; five injections/week for 5 weeks), different organs were ablated and processed for routine histopathological examination. As illustrated, haematoxylin–eosin staining did not reveal any significant macroscopic or microscopic tissue alterations in liver or kidney in any of the three experimental groups (non-injected, vehicle-injected or α5IA-treated mice). The same negative findings were observed following periodic acid-Schiff staining of the tissues (not illustrated). Examination of brain, hepatic and renal tissues under polarized light revealed the lack of abnormal crystals in mice receiving injections of α5IA. The size and distribution of urine crystals (not illustrated) appeared to be very similar in the different groups. Scale bar = 100 µm.

Following 5 weeks of chronic treatment with α5IA, various organs were collected and processed for routine histopathological examination. Haematoxylin–eosin (Figure 8) and periodic acid-Schiff stained sections (not shown) did not reveal any significant macroscopic nor microscopic tissue alterations in any of the three experimental groups (non-injected, vehicle-injected or α5IA-treated mice). In particular, examination of brain, hepatic and renal tissues under polarized light did not show the occurrence of abnormal crystals in mice that did receive injections of α5IA.

In summary, it appears that treatment with α5IA did not promote any significant liabilities as it did not induce convulsant/pro-convulsant activity nor affected locomotion and anxiety-related behaviours.

## Discussion

### α5IA restores cognitive dysfunction in Ts65Dn mice

In this study we demonstrated that treatment with α5IA largely alleviates the cognitive deficits of Ts65Dn mice. Indeed Ts65Dn mice receiving a single administration of α5IA increased their memory performance in the NOR task and behaved as α5-IA-treated euploid littermates. Furthermore, repeated α5IA treatment across training sessions in the MWM task allowed Ts65Dn mice to decrease their anomalous foraging behaviours, and to learn a fixed goal location with the same efficiency as euploid mice. Rescue of learning deficits in Ts65Dn mice by α5IA appeared to be specific since sensory functions in the MWM test or motivation to explore objects in the NOR task remained unchanged in this genotype and were not affected by α5IA treatment. These exciting findings provide, for the first time, important preclinical evidence for the hypothesis that release of GABAergic inhibition by α5 GABA-A benzodiazepine inverse agonists may improve cognitive function in DS individuals.

Treatment with GABA_A_ antagonists (e.g. pentylenetetrazol) was previously shown to rescue memory performances in Ts65Dn mice trained in the NOR task (Fernandez et al., 2007) and in the MWM task (Rueda et al., 2008). However, the use of GABA antagonists as well as of non-specific GABA-A benzodiazepine inverse agonists as therapeutic molecules has serious limitations because of their known adverse effects: convulsant, pro-convulsant and anxiogenic effects. The α5 GABA-A benzodiazepine inverse agonists, thanks to their unique pharmacological profile, are devoid of such liabilities (for a review see Atack, 2009). In the present study, we further show that Ts65Dn mice treated with α5IA did not display any alteration in their locomotor behaviour. More importantly and as opposed to treatments with pentylenetetrazol, Ts65Dn did not develop significant alterations of anxiety-related behaviours nor any convulsant or pro-convulsant activity. A putative renal toxicity of α5IA has been claimed in some reports (Atack, 2008; Merschman et al., 2005) because of the *in vivo* formation and crystallization of insoluble metabolites at extremely high dosages (240 mg/kg/day for 5 weeks). However, we did not find evidence of any anatomo-pathological lesions in mice chronically treated with α5IA at 5 mg/kg, the pharmacologically active dose (see Supplemental Text T1 for additional discussion).

From these observations it can be concluded that α5IA has a better therapeutic profile than GABA antagonists. Indeed the first successful use of α5IA as a cognitive enhancer candidate in human subjects has been recently published (Nutt et al., 2007) affirming its good safety and tolerability.

### α5IA effects on acquisition and retrieval of memories

In addition to its therapeutic effects in Ts65Dn mice, α5IA displayed some promnesic action in euploid mice trained in short-term memory tasks using the NOR or DMTP paradigms. Most studies investigating cognitive-enhancing properties of α5-specific GABA-A inverse agonists were indeed conducted in rodents trained in the DMTP test (Collinson et al., 2002; Dawson et al., 2006). However, in a spatial reference memory task requiring gradual memorization of an invariant goal location throughout trials and days (MWM task) we showed that α5IA largely facilitated the performance of Ts65Dn mice but not those of euploid mice. This underscores that α5IA, under non-pathological conditions, might have positive outcomes but only in specific (short-term memory) training conditions.

When evaluating the effects of α5IA on the retrieval of long-term (24 hours) spatial memory during the probe test of the MWM task, we showed that α5IA did not actually increase retention performance in either euploid or Ts65Dn mice. This indicates that α5IA mainly exerts its nootropic action during the acquisition of information but might be less potent in stimulating accurate retrieval of the previously formed memories. Collinson, Atack and colleagues suggested that GABA-A α5 inverse agonists could, under some circumstances, improve both the acquisition and the retrieval of spatial memories. However, they used memory paradigms based on short–intermediate retention intervals (15–180 min) that do not fully assess long-term recall (at least 24 hours post-acquisition) as usually performed during probe tests in spatial navigation tasks (Atack et al., 2006; Collinson et al., 2006).

Altogether these studies suggest that α5IA stimulates short-term memories in normal and cognitively impaired mice, likely through a modulation of the attentional–working memory process. In addition, gradual learning across training sessions, as evaluated in the MWM task, can also be potentiated by α5IA in Ts65Dn mice but the effects are less pronounced in euploid mice displaying high learning proficiencies in this task. Finally, the stabilization and late recall of reference memories do not appear to be impacted by α5IA treatment.

### Putative mechanisms of action of α5IA in normal and diseased brain

In close association with an enhancement of cognitive proficiency, we showed that treatment with α5IA also increased immediate early gene products (Fos protein levels) following a behavioural stimulation that mimics a learning episode (encoding of new information). Increased Fos immunoreactivity was observed in all of the sampled brain areas involved in recognition memory (posterior cingulate and perirhinal cortices, pyramidal cell layer of the hippocampus) but not at the level of the dentate gyrus. This latter observation was expected as the dentate gyrus is a sector of the hippocampus that displays only low concentrations of GABA_A_ α5 receptors (Pirker et al., 2000; Sperk et al., 1997). Paucity of targets might hence explain the local lack of drug-induced increased neuronal activity. Importantly we did not find any differences between euploid and Ts65Dn mice in terms of Fos immunoreactivity levels. The absence of a genotype effect under vehicle conditions underscores that Ts65Dn mice did not sustain an overall pattern of reduced neuronal activity, at least during the exploration–memorization of a new environment. Following drug administration, both genotypes displayed significant (and comparable) increases in the levels of neuronal activity markers. This potentiation of brain activity during acquisition of new information might therefore be the substratum of the ‘general’ promnesic effects of α5IA that should be independent of the disease status.

While we showed that Ts65Dn mice displayed similar levels of brain activity as euploid mice, it is known from the literature that these mice concurrently develop synaptic plasticity anomalies as exemplified by impaired LTP (Siarey et al., 1997). Reduction of synaptic plasticity in Ts65Dn mice is observed in the absence of any notable changes in the general properties of excitatory synaptic transmission (Kleschevnikov et al., 2004). Importantly these LTP deficits can be rescued following release of the GABAergic inhibitory transmission by means of picrotoxin (Kleschevnikov et al., 2004). In parallel it has been shown recently that α5 GABA-A inverse agonists, including the drug used in the present study, potentiate LTP in mouse hippocampal slices (Ballard et al., 2009; Dawson et al., 2006) and it can be postulated that these drugs likely have the potential to reverse LTP deficits and concomitantly to improve cognition in Ts65Dn mice.

In conclusion, we have demonstrated that an α5-selective GABA-A inverse agonist can restore cognitive function (short-term recognition memory and spatial learning) in a mouse model of DS. Our results strengthen the hypothesis that modifying the GABAergic-mediated balance between excitatory and inhibitory neurotransmission can efficiently alleviate cognitive impairments in preclinical models of DS. The exact mechanism of action of α5IA remains to be clarified, but might involve potentiation of neuronal activity and of synaptic plasticity of neural networks.

α5IA, because of its lack of convulsant or anxiogenic effects, has a more favourable therapeutic profile than other GABAergic drugs such as pentylenetetrazol. Also we did not detect any toxicity of α5IA following repeated injections. The first successful use of α5IA as a cognitive enhancer for blocking alcohol's amnestic activity in human subjects has indeed been published, confirming it as safe and well tolerated (Nutt et al., 2007). The excellent safety profile of α5IA and of similar recently developed compounds will undoubtedly facilitate their clinical investigation in individuals with DS.

## References

[bibr1-0269881111405366] Atack JR (2008) GABA(A) receptor subtype-selective efficacy: TPA023, an alpha2/alpha3 selective non-sedating anxiolytic and alpha5IA, an alpha5 selective cognition enhancer. CNS Neurosci Ther 14: 25–35 18482097 10.1111/j.1527-3458.2007.00034.x PMC6494020

[bibr2-0269881111405366] Atack JR (2009) Preclinical and clinical pharmacology of the GABA(A) receptor alpha5 subtype-selective inverse agonist alpha5IA. Pharmacol Ther in press 10.1016/j.pharmthera.2009.09.001 19770002

[bibr3-0269881111405366] Atack JR Bayley PJ Seabrook GR (2006) L-655, 708 enhances cognition in rats but is not proconvulsant at a dose selective for alpha5-containing GABAA receptors. Neuropharmacology 51: 1023–1029 17046030 10.1016/j.neuropharm.2006.04.018

[bibr4-0269881111405366] Ballard TM Knoflach F Prinssen E (2009) RO4938581, a novel cognitive enhancer acting at GABA(A) alpha5 subunit-containing receptors. Psychopharmacology (Berl) 202: 207–223 18936916 10.1007/s00213-008-1357-7

[bibr5-0269881111405366] Best TK Siarey RJ Galdzicki Z (2007) Ts65Dn, a mouse model of Down syndrome, exhibits increased GABAB-induced potassium current. J Neurophysiol 97: 892–900 17093127 10.1152/jn.00626.2006

[bibr6-0269881111405366] Collinson N Atack JR Laughton P (2006) An inverse agonist selective for alpha5 subunit-containing GABAA receptors improves encoding and recall but not consolidation in the Morris water maze. Psychopharmacology (Berl) 188: 619–628 16633803 10.1007/s00213-006-0361-z

[bibr7-0269881111405366] Collinson N Kuenzi FM Jarolimek W (2002) Enhanced learning and memory and altered GABAergic synaptic transmission in mice lacking the alpha 5 subunit of the GABAA receptor. J Neurosci 22: 5572–5580 12097508 10.1523/JNEUROSCI.22-13-05572.2002 PMC6758233

[bibr8-0269881111405366] Costa AC Stasko MR Schmidt C (2010) Behavioral validation of the Ts65Dn mouse model for Down syndrome of a genetic background free of the retinal degeneration mutation Pde6b(rd1). Behav Brain Res 206: 52–62 19720087 10.1016/j.bbr.2009.08.034 PMC2783207

[bibr9-0269881111405366] Dawson GR Maubach KA Collinson N (2006) An inverse agonist selective for alpha5 subunit-containing GABAA receptors enhances cognition. J Pharmacol Exp Ther 316: 1335–1345 16326923 10.1124/jpet.105.092320

[bibr10-0269881111405366] Demas GE Nelson RJ Krueger BK (1996) Spatial memory deficits in segmental trisomic Ts65Dn mice. Behav Brain Res 82: 85–92 9021073 10.1016/s0166-4328(97)81111-4

[bibr11-0269881111405366] Escorihuela RM Fernandez-Teruel A Vallina IF (1995) A behavioral assessment of Ts65Dn mice: a putative Down syndrome model. Neurosci Lett 199: 143–146 8584244 10.1016/0304-3940(95)12052-6

[bibr12-0269881111405366] Faure A Verret L Bozon B (2009) Impaired neurogenesis, neuronal loss, and brain functional deficits in the APPxPS1-Ki mouse model of Alzheimer's disease. Neurobiol Aging in press 10.1016/j.neurobiolaging.2009.03.009 19398247

[bibr13-0269881111405366] Fernandez F Morishita W Zuniga E (2007) Pharmacotherapy for cognitive impairment in a mouse model of Down syndrome. Nat Neurosci 10: 411–413 17322876 10.1038/nn1860

[bibr14-0269881111405366] Hoelter SM Dalke C Kallnik M (2008) “Sighted C3H” mice—a tool for analysing the influence of vision on mouse behaviour? Front Biosci 13: 5810–5823 18508624 10.2741/3118

[bibr15-0269881111405366] Janus C Welzl H Hanna A (2004) Impaired conditioned taste aversion learning in APP transgenic mice. Neurobiol Aging 25: 1213–1219 15312967 10.1016/j.neurobiolaging.2003.11.007

[bibr16-0269881111405366] Kleschevnikov AM Belichenko PV Villar AJ (2004) Hippocampal long-term potentiation suppressed by increased inhibition in the Ts65Dn mouse, a genetic model of Down syndrome. J Neurosci 24: 8153–8160 15371516 10.1523/JNEUROSCI.1766-04.2004 PMC6729789

[bibr17-0269881111405366] Menendez M (2005) Down syndrome, Alzheimer's disease and seizures. Brain Dev 27: 246–252 15862185 10.1016/j.braindev.2004.07.008

[bibr18-0269881111405366] Merschman SA Rose MJ Pearce GE (2005) Characterization of the solubility of a poorly soluble hydroxylated metabolite in human urine and its implications for potential renal toxicity. Pharmazie 60: 359–363 15918586

[bibr19-0269881111405366] Nutt DJ Besson M Wilson SJ (2007) Blockade of alcohol's amnestic activity in humans by an alpha5 subtype benzodiazepine receptor inverse agonist. Neuropharmacology 53: 810–820 17888460 10.1016/j.neuropharm.2007.08.008

[bibr20-0269881111405366] Pirker S Schwarzer C Wieselthaler A (2000) GABA(A) receptors: immunocytochemical distribution of 13 subunits in the adult rat brain. Neuroscience 101: 815–850 11113332 10.1016/s0306-4522(00)00442-5

[bibr21-0269881111405366] Pueschel SM Louis S McKnight P (1991) Seizure disorders in Down syndrome. Arch Neurol 48: 318–320 1825777 10.1001/archneur.1991.00530150088024

[bibr22-0269881111405366] Reeves RH Garner CC (2007) A year of unprecedented progress in Down syndrome basic research. Ment Retard Dev Disabil Res Rev 13: 215–220 17910083 10.1002/mrdd.20165

[bibr23-0269881111405366] Reeves RH Irving NG Moran TH (1995) A mouse model for Down syndrome exhibits learning and behaviour deficits. Nat Genet 11: 177–184 7550346 10.1038/ng1095-177

[bibr24-0269881111405366] Rueda N Florez J Martinez-Cue C (2008) Chronic pentylenetetrazole but not donepezil treatment rescues spatial cognition in Ts65Dn mice, a model for Down syndrome. Neurosci Lett . 10.1016/j.neulet.2007.12.039 18226451

[bibr25-0269881111405366] Sherman SL Allen EG Bean LH (2007) Epidemiology of Down syndrome. Ment Retard Dev Disabil Res Rev 13: 221–227 17910090 10.1002/mrdd.20157

[bibr26-0269881111405366] Siarey RJ Stoll J Rapoport SI (1997) Altered long-term potentiation in the young and old Ts65Dn mouse, a model for Down syndrome. Neuropharmacology 36: 1549–1554 9517425 10.1016/s0028-3908(97)00157-3

[bibr27-0269881111405366] Sperk G Schwarzer C Tsunashima K (1997) GABA(A) receptor subunits in the rat hippocampus I: immunocytochemical distribution of 13 subunits. Neuroscience 80: 987–1000 9284055 10.1016/s0306-4522(97)00146-2

[bibr28-0269881111405366] Sternfeld F Carling RW Jelley RA (2004) Selective, orally active gamma-aminobutyric acidA alpha5 receptor inverse agonists as cognition enhancers. J Med Chem 47: 2176–2179 15084116 10.1021/jm031076j

[bibr29-0269881111405366] Sur C Fresu L Howell O (1999) Autoradiographic localization of alpha5 subunit-containing GABAA receptors in rat brain. Brain Res 822: 265–270 10082908 10.1016/s0006-8993(99)01152-x

[bibr30-0269881111405366] Veall RM (1974) The prevalance of epilepsy among mongols related to age. J Ment Defic Res 18: 99–106 4281033 10.1111/j.1365-2788.1974.tb01224.x

[bibr31-0269881111405366] Wisden W Laurie DJ Monyer H (1992) The distribution of 13 GABAA receptor subunit mRNAs in the rat brain. I. Telencephalon, diencephalon, mesencephalon. J Neurosci 12: 1040–1062 1312131 10.1523/JNEUROSCI.12-03-01040.1992 PMC6576059

[bibr32-0269881111405366] Wiseman FK Alford KA Tybulewicz VL (2009) Down syndrome–recent progress and future prospects. Hum Mol Genet 18: R75–R83 19297404 10.1093/hmg/ddp010 PMC2657943

[bibr33-0269881111405366] Wishart JG Willis DS Cebula KR (2007) Collaborative learning: comparison of outcomes for typically developing children and children with intellectual disabilities. Am J Ment Retard 112: 361–374 17676960 10.1352/0895-8017(2007)112[0361:CLCOOF]2.0.CO;2

